# Biofilms in clinical infection: pathophysiology, diagnosis, and the evolving therapeutic landscape

**DOI:** 10.1128/jcm.01042-25

**Published:** 2025-12-17

**Authors:** Aglaia Domouchtsidou, Petros Ioannou, Alexandra Lianou, Konstantina A. Tsante, Deny Tsakri, Elli Bonova, Stella Baliou, Sotirios P. Fortis, Anastasios E. Chaldoupis, George Samonis, Christos Koutserimpas, Rozeta Sokou, Dimitrios V. Papadopoulos, Andreas G. Tsantes

**Affiliations:** 1 Microbiology Department, "Saint Savvas" Oncology Hospital, Athens, Greece; 2 School of Medicine, University of Crete 37778, Heraklion, Greece; 3 Neonatal Intensive Care Unit, "Agios Panteleimon" General Hospital of Nikea, Piraeus, Greece; 4 Department of Biomedical Sciences, University of West Attica, Athens, Greece; 5 Department of Microbiology, Medical School, National and Kapodistrian University of Athens 68993, Athens, Greece; 6 Department of Biomedical Sciences, Humanitas University, Pieve Emanuele 437807, Milan, Italy; 7 Laboratory of Toxicology, School of Medicine, University of Crete 37778, Heraklion, Greece; 8 Laboratory of Reliability and Quality Control in Laboratory Hematology (HemQcR), Department of Biomedical Sciences, School of Health & Caring Sciences, University of West Attica 523391, Athens, Greece; 9 Laboratory of Haematology and Blood Bank Unit, School of Medicine, "Attiko" Hospital, National and Kapodistrian University of Athens 68989, Athens, Greece; 10 First Department of Medical Oncology, Metropolitan Hospital of Neon Faliron, Athens, Greece; 11 School of Rehabilitation Health Sciences, University of Patras 37795, Patras, Greece; 12 Neonatal Department, Aretaieio Hospital, National and Kapodistrian University of Athens 68993, Athens, Greece; 13 Second Department of Orthopaedic Surgery, School of Medicine, Konstantopouleio General Hospital, National and Kapodistrian University of Athens 68989, Athens, Greece; Medical College of Wisconsin, Milwaukee, Wisconsin, USA

**Keywords:** biofilm, structure, pathophysiology, diagnostic modalities, treatment strategies

## Abstract

Biofilms are structured communities of microorganisms encased in a self-produced polymeric matrix that typically adhere to surfaces. Recent research, however, has revealed that non-attached aggregates share many common traits with the surface-dependent biofilms. This mode of bacterial growth provides enhanced protection against antibiotics and resistance to host immune defenses. Biofilms require higher antibiotic concentrations than those needed to inhibit planktonic bacteria, necessitating prolonged high-dose and combination therapies to achieve effective eradication. This increased resistance is attributed to multiple factors, including the protective extracellular matrix, reduced metabolic activity of bacteria within the biofilm, and also the ability of bacterial genomes to rapidly adjust in response to environmental changes. Diagnostic modalities such as sonication, tissue culture, and polymerase chain reaction-based assays currently dominate clinical diagnostics of biofilm infections due to their practicality, cost-effectiveness, and proven reliability. Recent research has led to innovative treatment strategies that target biofilm structure, enhance drug delivery, and modulate host-pathogen interactions. This review summarizes our current knowledge of biofilm formation, explores the current techniques for detecting microbial biofilms, and discusses future perspectives for advancing diagnostic and therapeutic strategies.

## INTRODUCTION

Bacteria can grow in two primary modes: the planktonic (free-living) mode and the sessile mode (1). In the planktonic mode, bacteria exist as single, free-swimming cells, often in a liquid environment. This mode allows them to move and colonize new niches but makes them more vulnerable to environmental stresses, including antimicrobial agents. In contrast, the sessile mode involves bacteria forming biofilms, which are structured communities of cells encased in a self-produced polymeric matrix that adheres to surfaces (2, 3).

A biofilm can consist of one microbial species or can be a collection of different microorganisms, such as bacteria, fungi, and protozoa, along with viruses, coexisting in the polysaccharide matrix known as extracellular polymeric substance (EPS) (4, 5). This matrix is produced by the microorganisms and is primarily composed of polysaccharides, proteins, extracellular DNA, and nucleic acids (6, 7). While not the sole mediator of adhesion, the matrix also protects against environmental stressors and antibiotics, contributing to the high antibiotic resilience of biofilms (8–10).

Biofilms play a critical role in the persistence and resistance of microbial infections, particularly those associated with medical devices like catheters, prosthetics, and ventilators (11). Infection rates are estimated to be around 2% for joint prostheses and breast implants, 4% for mechanical heart valves, pacemakers, and defibrillators, and as high as 40% for ventricular assist devices (12).

This review summarizes our current knowledge of biofilm formation, focuses on the clinical relevance of biofilms in chronic and device-associated infections, critically examines the limitations of current methodologies, and evaluates emerging treatment regimens.

## BIOFILM LIFE CYCLE

**Fig 1:**
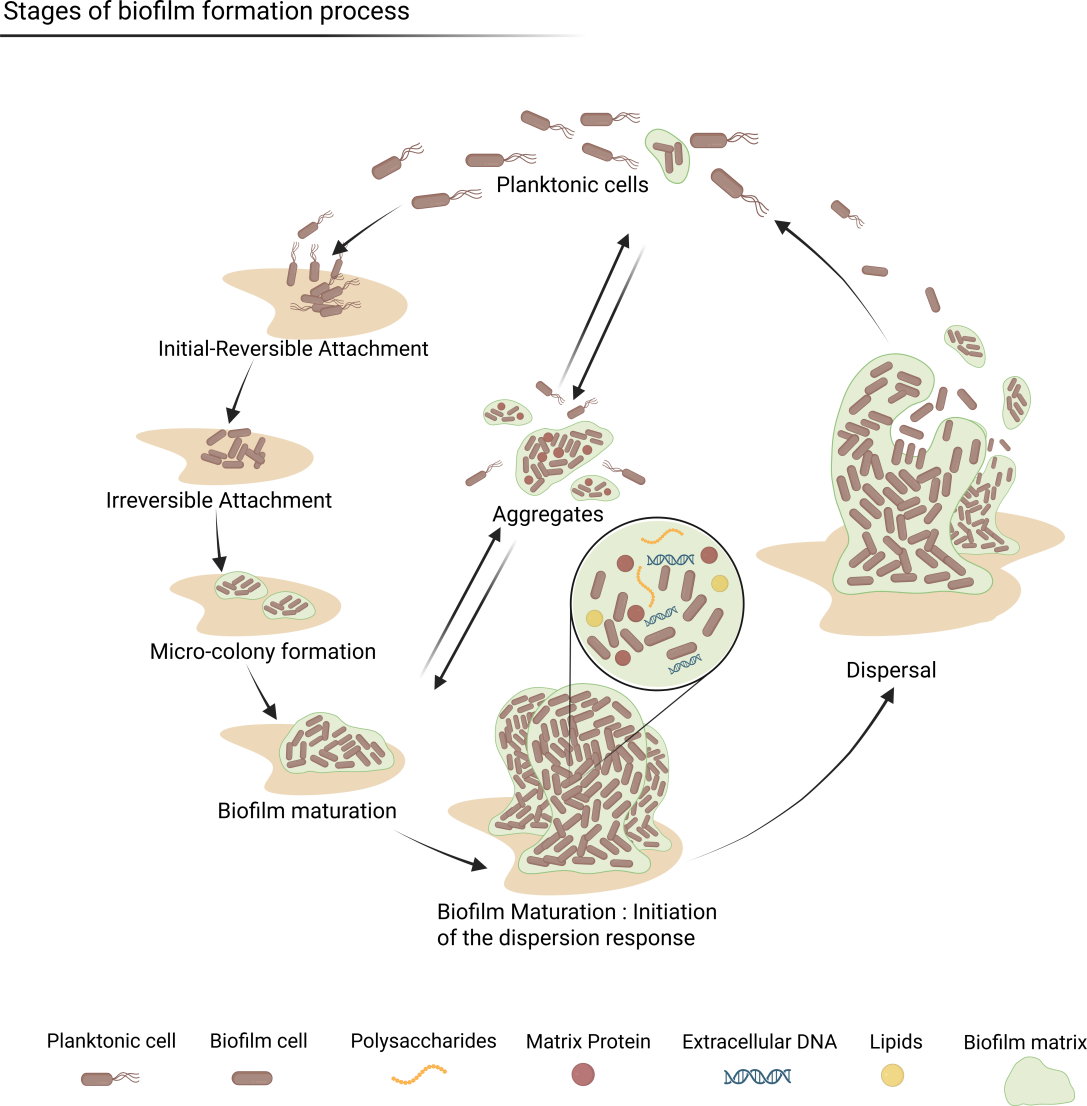
Stages of biofilm formation. Visual presentation of the transition from free-living bacteria (planktonic cells) to structured microbial communities. The process includes initial reversible attachment, irreversible attachment, micro-colony formation, biofilm maturation, and dispersal. Key components of the biofilm matrix include the following: polysaccharides, matrix protein, extracellular DNA, and lipids. The surrounding structural environment is referred to as the biofilm matrix. Created in BioRender (2025) https://BioRender.com/isv08k5.

While it is now broadly recognized that nearly all bacterial species form biofilms, the earliest insights into the biofilm life cycle came from studies on *Pseudomonas aeruginosa* (13–15). These studies led to the current five-step model: (i) initial contact and attachment to a surface (reversible attachment), (ii) irreversible attachment, (iii) formation of microcolonies, (iv) maturation, and (v) detachment/dispersion (16, 17) (Fig. 1). Each step involves intercellular communication and is guided by a set of unique gene expression profiles, which adapt to environmental signals (7, 18).

Recent research has revealed that attachment to a surface is not always a prerequisite in biofilm formation (19) and that non-attached aggregates share many common traits with the surface-dependent biofilms, such as similar growth rates and matrix consistency (20, 21). Sauer et al. proposed a revised model including the three major stages of biofilm development—aggregation, growth, and disaggregation—occurring independently of biotic or abiotic surfaces (22).

The stages leading to biofilm formation will be discussed below.

## STAGES

### Biofilm initiation

Biofilm development typically begins with bacterial adhesion to a surface. However, in accordance with the revised model of biofilm formation, this step is not universal, as non-attached, “free-floating” biofilm aggregates can also form in suspension (22). Biofilm formation begins when planktonic bacterial cells sense favorable conditions, for example, surface chemistry, presence of nutrients, suitable temperature, or a particular pH (23–25). These conditions initiate biofilm formation through multiple regulatory pathways. Evidence indicates that one of the most significant shifts is a rise in cyclic dimeric guanosine monophosphate (c-di-GMP)—a signaling molecule that promotes the adhesion and extracellular matrix production while suppressing motility. Through this regulation, c-di-GMP orchestrates, especially in gram-negative bacteria, the transition between the motile planktonic state and the stationary, biofilm-associated way of life (18, 26).

In surface-associated biofilms, cells experience transient interactions with the attached surface, which can result in reversible attachment (27). After making initial contact, gram-negative bacteria utilize force-enhancing structures (either flagella or type IV pili) to move across the surface in two dimensions until they encounter other bacteria, leading to irreversible attachment and the formation of microcolonies (25, 28–30). In contrast to motile microbes, initial attachment to abiotic surfaces in gram-positive bacteria (e.g., *Staphylococcus aureus*, *Staphylococcus epidermidis*) is mainly passive, relying on hydrophobic and electrostatic interactions (7). Subsequently, tighter adhesion to biotic or abiotic substrates is mediated by surface proteins, such as MSCRAMMs (7, 31). This distinction is clinically important, as gram-positive bacteria are major contributors to biofilm-related device infections (32, 33). MSCRAMMs—microbial surface components recognizing adhesive matrix molecules—bind host extracellular-matrix proteins (fibrinogen, fibronectin, collagen) that rapidly cover indwelling device surfaces (34, 35).

### Biofilm formation and maturation

Microcolony formation results from the cell division and recruitment of planktonic cells or by the incorporation of newly formed bacterial clusters. Attachment to a surface triggers alterations in gene expression and the production of exopolysaccharides, which constitute the matrix (36).

From the moment the cell density surpasses a certain threshold, a mechanism that enables the communication between cells is activated, called quorum-sensing (QS) system (37). QS allows cells to coordinate their behavior via small signaling molecules called autoinducers (38, 39).

Following its initial establishment, the biofilm undergoes a maturation phase. During this stage, the initially single-layered attached cells accumulate into multiple layers, increasing the overall thickness and shifting the arrangement from two- to three-dimensional, with 50%–90% consisting of EPS (9, 23, 40).

### Biofilm dispersion

Biofilm dispersion is the final stage of biofilm development and can be either a passive or an active event. Passive dispersal occurs when cells detach due to external forces such as fluid shear, whereas active dispersal occurs when bacteria initiate their own detachment from the biofilm (41, 42). This process plays a critical role in bacterial pathogenicity since it facilitates the spread of infections in clinical settings (43). Stressful situations within the biofilm (e.g., nutrient depletion, oxygen gradients), but also stressors in the surrounding environment, can act as cues for disassembly, prompting bacteria to detach from the biofilm and transition back to a planktonic state (2, 44–47).

Upon signals that initiate dispersal, the bacterial cells start to produce extracellular enzymes that degrade either the biofilm matrix or the substrate on which the biofilm has attached (48, 49). As with biofilm formation, the dissemination of the mature biofilm is orchestrated by QS (50, 51).

## ΒIOFILM-RELATED INFECTIOUS DISEASES

The number of infections linked to biofilm formation continues to increase each year, prompting ongoing refinement of the term “biofilm” to incorporate emerging insights, such as the fact that pathogenic biofilm aggregates can form without adhering to a surface. Examples of recently identified non-adhering biofilm infections include *Mycobacterium abscessus* complex pulmonary infections in cystic fibrosis (CF) patients with end-stage lung disease (52) and *Borrelia burgdorferi* aggregates in infected human skin lesions (53). The list of infections associated with surface-adhering biofilms also expands, with chronic fungal onychomycosis and *P. aeruginosa* respiratory infection in patients with bronchiectasis recently being recognized as biofilm infectious diseases (54, 55). In *E. coli* urinary tract infections (UTI), intracellular bacterial communities (IBCs) form within urothelial cells, constituting an intrinsic biofilm phase that contributes to persistence and recurrence (56, 57). We will refer to some of the most extensively studied biofilm-associated infections.

### Infective endocarditis

Research has established the central role of biofilm formation in the pathogenesis of IE, especially in cases involving prosthetic materials (58–60). Infective endocarditis (IE) has a high mortality rate, with one-third of patients dying within the first year after infection (61, 62). Prompt identification of the specific microbial cause and administration of targeted antimicrobial treatment are integral for achieving the best outcome in patient care. Blood cultures remain the gold standard for identifying cultivable organisms that cause endocarditis, including *Candida* species (63). *Staphylococci*, *streptococci,* and *enterococci* are the most commonly isolated microorganisms. *Staphylococcus aureus* and coagulase-negative staphylococci (CoNS), such as *Staphylococcus epidermidis,* are known for their ability to form resilient biofilms (7). Especially for *S. epidermidis*, which, in contrast to *S. aureus*, lacks the variety of virulence mechanisms, biofilm formation is crucial to exert its pathogenicity (64). In prosthetic valve endocarditis and pacemakers’ infections, the most isolated microorganisms are CoNS, whereas *S. aureus* is the most frequent cause of IE on native valves (65, 66). The aortic and the mitral valves are the most commonly affected valves, with microbes often forming biofilms that lead to the formation of cardiac valve vegetations recalcitrant to antibiotic therapy (58, 62, 67, 68).

Gram-negative bacilli, fungi, and some difficult-to-cultivate microorganisms, such as *Coxiella burnetii*, account for less than 10% of IE cases (66). Fastidious bacteria and fungi (such as *Coxiella burnetii*, *Bartonella* spp., *Aspergillus* spp., *Mycoplasma pneumoniae*, *Brucella* spp.) must be considered after culture-negative results and necessitate further specific serological and molecular diagnostics (69). The modified Duke criteria, which have long been a fundamental diagnostic tool, were updated in 2023 by the European Society of Cardiology (ESC), with the revisions being introduced as the Modified diagnostic criteria of IE, in the 2023 ESC Guidelines for the management of endocarditis (69). The significance of biofilm formation is evident in that when antibiotic treatment fails, approximately 50% of IE cases require surgical intervention to manage the infection and restore heart function (70).

### Chronic wound infections

Biofilms are deeply implicated in the persistence and chronicity of wound infections—they are thought to play a significant role in four out of five chronic wound infections (71). Chronic non-healing wounds are often linked to underlying conditions such as diabetes, older age, immunosuppression, and autoimmune diseases (72–74). In the context of chronic wounds—such as diabetic foot ulcers, venous leg ulcers, and pressure injuries—biofilms establish themselves within necrotic tissue and wound exudate, where local hypoxia and impaired immune surveillance promote bacterial survival (75–77). The presence of biofilms in these environments contributes to sustained inflammation, overexpression of proteases, and degradation of extracellular matrix components, all of which delay granulation and epithelialization (78–81).

The microbial communities in wound biofilms are typically polymicrobial and spatially organized within the wound bed (82–84). Among the most frequently implicated species are *Staphylococcus aureus* and *Pseudomonas aeruginosa*, which persistently colonize chronic wounds and exhibit synergistic interactions that enhance their virulence and resistance (85, 86). *S. aureus* is known to induce host cell apoptosis and modulate local cytokine profiles, while *P. aeruginosa* secretes proteolytic enzymes and pyocyanin, disrupting tissue repair processes (87, 88). Additional contributors include CoNS, *Enterococcus* spp., *Klebsiella pneumoniae*, *Escherichia coli*, and anaerobic species such as *Bacteroides* and *Prevotella* (86, 89–92). These biofilm communities contribute to pathological wound stasis by interfering with leukocyte recruitment and diminishing macrophage phagocytic function (93, 94). Furthermore, the interactions among constituent microbes often lead to cooperative biofilm architecture, horizontal gene transfer, and the emergence of multidrug resistance, complicating infection control and prolonging tissue injury (95, 96).

Nevertheless, even though biofilms are frequent in chronic wounds, they do not necessarily impair healing (97). Their contribution to chronicity is still being defined and should be interpreted in light of host factors and the wound environment. In 2025, the International Wound Infection Institute emphasized therapeutic cleansing, debridement, and wound dressings as essential to prevent biofilm formation (98).

### Cystic fibrosis

It was first in 1980 that Nils Høiby observed a connection between persistent lung infections and *Pseudomonas aeruginosa* bacterial aggregates in CF patients (99). CF is a hereditary genetic disease. It is caused by a mutation in the CFTR gene (Cystic Fibrosis Transmembrane Conductance Regulator), which leads to the production of abnormally thick and viscous mucus, primarily affecting the lungs but also causing a wide range of clinical sequelae. The disease is considered a biofilm-predisposing condition (100). Biofilms of *P. aeruginosa* have been detected in lung tissue and sputum of patients with CF (101–103). *P. aeruginosa* biofilms that are formed in sputum can induce endobronchiolitis without attaching to the nearby epithelial cells or spreading hematogenously to other organs. *Staphylococcus aureus* and *Haemophilus influenzae* are also well-studied pathogens in the context of CF and usually colonize the airways of CF patients already in early life (104, 105). Apart from different opportunistic bacteria, fungi also contribute to the diverse microbial populations that constitute the biofilms formed in the CF lungs (105, 106). These biofilms pose major diagnostic and therapeutic challenges, leading experts to frequent revisions of the best treatment strategies in the combat of persistent respiratory infections in CF patients (107, 108). A combination of nebulized and intravenous antibiotic therapy is considered to be necessary in chronically infected *P. aeruginosa* patients (109).

### Medical device-associated infections

Apart from prosthetic heart valves and pacemakers, biofilms can form on a wide range of implanted devices, such as joint prostheses, spinal implants, central venous catheters, urinary catheters, ventilators, and dental prostheses. Table 1 shows a comprehensive list of medical device-associated biofilm infections. The most common pathogenic bacteria implicated in biofilm infections related to medical devices are *E. coli*, *K. pneumoniae*, *P. mirabilis*, *P. aeruginosa*, *A. baumannii*, *S. aureus*, *S. epidermidis, S. pneumoniae,* and *E. faecalis* (32, 110). Significant efforts are being dedicated to developing effective prevention strategies. Ensuring aseptic conditions during surgery and device placement, reducing bacterial adhesion by coating devices with antimicrobials, such as silver or antibiotics, developing biofilm-resistant materials, and administering prophylactic antibiotics before implantation are the strategies currently used to reduce the risk of biofilm formation.

**TABLE 1:** Biofilm-associated infections on medical devices

Implant location	Device-related infections	References
Bones	Orthopedic implants Prosthetic joints	Kuik et al. (111), Doub et al. (112), Gbejuade et al*.* (113), Tsantes et al. (114), Zhang et al. (115)
Brain	External ventricular drainage Cerebrospinal shunts	Stoodley et al. (116), Ramírez et al. (117), Roujansky et al. (118), Fux et al. (119)
Circulatory system	Central venous catheters Peripheral vascular catheters Prosthetic cardiac valves Vascular grafts Pacemakers Defibrillators	Passerini et al. (120), Niemann et al. (121), Kouijzer & Noordermeer et al. (122), Makis & Stern et al. (123)
Eye	Contact lenses Intraocular lenses	Buzalewicz et al. (124), Voinescu et al. (125), Yi & Sun et al. (126), Kodjikian et al. (127), Kishimoto et al. (128), Mazoteras et al. (129)
Respiratory system	Endotracheal tubes Tracheostomy tubes Voice prostheses	Maldiney et al. (110), Diaconu et al. (130), Raveendra et al. (131), Ścibik et al. (132), Spałek et al. (133)
Skin and soft tissues	Breast implants Penile implants Tissue fillers	Whitfield et al. (134), Foppiani et al. (135), Leong et al. (136), Silverstein et al. (137), Zhang et al. (138)
Teeth	Dental implants	Dieckow et al. (139), Fürst et al. (140)
Reproductive system	Intrauterine devices Fertility control devices	Jeyarajan et al. (141), Auler et al. (142), Hardy et al. (143), Carson et al. (144)
Urinary tract	Urinary catheters Ureteral stents	Rubi et al. (145), Amado et al. (146)

## CURRENT METHODS USED FOR DIAGNOSIS

Although recent studies have demonstrated that biofilms can also be present in acute infections, they are mostly associated with chronic infections (147). Unlike acute infections, biofilm infections often follow a progressive course with persistent, nonspecific clinical manifestations that may resemble noninfectious inflammatory processes, thus delaying clinical detection and diagnosis (100, 148, 149). The European Society of Clinical Microbiology and Infectious Diseases (ESCMID) released a guideline in 2014 for the diagnosis of infections related to biofilms, with a focus on key clinical and laboratory parameters (100). Clinically, patients may present with low-grade inflammatory reactions (swelling, redness, pain, loss of function) and occasionally low-grade fever. Medical history may reveal predisposing conditions for biofilm formation, such as implanted medical devices or CF. Furthermore, biofilm infection may present as persistent (of more than 7 days), recalcitrant to antibiotic treatment, or may show signs of recurrence after the treatment is stopped.

Diagnosis involves recognizing biofilm-driven infection and, once suspected, defining the causative pathogen(s); the methods summarized here (and in Table 2) address both aims, with many non-biofilm-specific assays still being valuable elements of the diagnostic armamentarium for biofilm-containing samples. With regard to the laboratory evaluation of a patient who developed a biofilm-related infection, the choice of laboratory technique depends on the location of the suspected biofilm and the type of sample available for analysis (150). There are three types of samples that can be analyzed for diagnosis of biofilm-related infections: it can be a tissue sample including the biofilm biomass, an implant device onto which the biofilm has formed, or a fluid sample such as blood, sputum, or wound exudate. Since biofilms are defined by bacterial aggregates attached to a surface, direct examination of tissue samples or infected materials, particularly in cases involving implanted devices, is the preferred approach. However, in many cases, direct sampling through procedures like biopsies or implant removal is not feasible, as the potential risks may outweigh the immediate clinical benefits. When such invasive methods are not appropriate, evaluation of fluid samples—such as blood, sputum, urine, synovial fluid, or wound exudate—is recommended (100). Swab sampling is generally discouraged due to its high likelihood of yielding false-negative results, partly because biofilms adhere strongly to host tissues (100, 151). To improve diagnostic accuracy, multiple or repeated sampling is advised to minimize the risk of missed detections.

**TABLE 2:** Main diagnostic modalities in biofilm-related infections^*b*^

Laboratory evaluation	Diagnostic modalities	Clinical utility	Limitations
Direct examination/microscopic evidence	Basic light microscopy	Identifies inflammatory cells, bacteria, and components of the biofilm Low cost Often the first step	Limited magnification/ resolution Low pathogen density in the sample can reduce diagnostic sensitivity.
	SEM	Provide high magnification/resolution images of the biofilm morphology	Limited to specialized laboratories High cost and technical expertise are needed
	CLSM	Can differentiate dead vs. live pathogens when combined with fluorescent probes and stains	Limited to specialized laboratories High cost and technical expertise are needed
Culture-based pathogen identification^*a*^	Tissue culture Fluid culture Implant culture	Gold standard	Does not diagnose a biofilm; detects growth only Invasive procedure for tissue/implant culture Lower sensitivity of the fluid culture
	Sonicated fluid culture	Sonication enhances recovery of biofilm-associated microbes	Does not diagnose a biofilm; detects growth only Higher rate of false positives due to contamination during processing
Genetic sequencing techniques	PCR^*a*^	16S rRNA PCR detects a gene present in all bacteria 16S rRNA PCR identifies bacterial species Improved sensitivity on implant sonicated fluid or tissue samples	Does not diagnose a biofilm; detects DNA only (viability unresolved) Cannot detect multiple organisms simultaneously Single-gene target or too short a 16S amplicon may not sufficiently resolve species identification Availability of primers and reference databases
	NGS	Sequences all genes, detecting multiple organisms simultaneously Provides information on resistance genes Helpful in cases of culture-negative and polymicrobial infections, or when previous antibiotics have been used	High cost and need for technical expertise Complex sample preparation Prone to false positives due to environmental DNA or contamination False negatives due to insensitive chemistry, low-throughput, or off-target amplification
	Transcriptomics	Genetic sequencing of mRNA Provide additional information on the functionality and activity of the involved pathogens	Currently used in research settings
Proteomics	MALDI-TOF mass spectrometry	Analysis of proteins in a sample through mass spectrometry Provides rapid species-level identification	Performed on isolated colonies Low sensitivity in polymicrobial infections Should be best used alongside, not instead of, conventional culture methods

^*a*^ Interpretation caveat: Culture and PCR do not diagnose a biofilm. Low quantitative results (low CFU or low PCR load) may reflect planktonic contamination—often gram-positive skin commensals—rather than infection.

^*b*^ SEM, scanning electron microscopy; CLSM, confocal laser scanning microscopy; PCR, polymerase chain reaction; NGS, next-generation sequencing; MALDI-TOF, matrix-assisted laser desorption ionization time-of-flight; CFU, colony-forming units.

The methods most widely used in clinical practice to diagnose biofilms include direct examination/microscopy, culture-based techniques, genetic sequencing techniques, and protein analysis (proteomics/metabolomics). Additional studies, such as sample fluid analysis for white blood cell count and differential (percentage of polymorphonuclear neutrophils), may be helpful (Table 2). In clinical microbiology laboratories, direct visualization techniques such as basic light microscopy are usually the first step of the diagnostic approach. Basic light microscopy of fluid/tissue samples or prosthetic materials, paired with standard stains like the Gram stain, can help identify inflammatory cells, bacteria, and components of the biofilm (152). Light microscopy is a basic and low-cost method that can provide visual confirmation of pathogens’ presence. However, this imaging technique has limited magnification and resolution, which is insufficient to fully visualize the architecture of a biofilm. Moreover, its sensitivity to detect a bacterial biomass is limited since bacteria are often present in low density in tissue or explanted material, and the background tissue may obscure them. For more detailed and accurate visualization, advanced imaging techniques like scanning electron microscopy (SEM) and confocal laser scanning microscopy (CLSM) are among the most effective tools for examining biopsy samples (153–155). SEM can provide high-resolution images of the biofilm morphology. The two main SEM techniques that have been studied for biofilm evaluation include conventional SEM and variable pressure SEM (VP-SEM) (153). Conventional SEM requires complete dehydration of the sample, which can distort the biofilm’s 3D structure and remove the EPS matrix, while VP-SEM can image samples with residual moisture, providing a more accurate evaluation of the native biofilm architecture (156). CLSM is another advanced optical imaging technique that can provide high-resolution 3D images of biological specimens in real time. This imaging technique is helpful in modern biofilm research, especially when combined with fluorescent probes and stains (FM 1-43 stain, fluorescent *in situ* hybridization probes, etc.) that can label specific cell types, DNA, and proteins (157). In the context of *E. coli* UTI, CLSM of exfoliated urothelial cells can visualize IBCs (56). The main advantage of CLSM over SEM is that it can be used with viability dyes (e.g., SYTO, propidium iodide); thus, it can differentiate between viable vs. non-viable bacteria or between metabolically active vs. dormant cells. However, although in some laboratories, advanced imaging techniques are coupled with basic light microscopy, these methods are not commonly available for routine use and are predominantly applied in research settings, where detailed structural analysis of biofilms is required.

Cultivation of the obtained specimen (tissue, fluid, or medical device) for bacterial growth is currently the gold standard for diagnosis of biofilm-related infections. Although the culture of fluid samples does not require any invasive procedure as opposed to tissue or implant culture, its sensitivity is limited, since, in contrast to planktonic bacteria, the pathogens forming bacterial aggregates in biofilms are usually not circulating in the obtained fluid samples. The sensitivity of fluid culture varies depending on several factors, including prior antibiotic use, quality of sample handling, and the pathogen involved. Several studies have evaluated the pathogen detection rate of fluid cultures in biofilm-related infections. In orthopedics, the sensitivity of synovial fluid culture for diagnosing PJI has been reported to be 60%–80%, while in neurosurgery, the sensitivity of cerebrospinal fluid culture for biofilm-associated neurosurgical device infections is even lower, less than 60% (158). In the case of extracted medical devices, sonication has proven to be a valuable technique for improving microbial detection of cultures by using ultrasound waves to dislodge bacteria embedded within biofilms on medical devices. This method is widely used for diagnosing infections associated with prosthetic joints, cardiovascular implants, neurosurgical devices, and vascular grafts (159). A 2024 meta-analysis by Watanabe et al. systematically assessed the diagnostic performance of various culture methods in detecting periprosthetic joint infections (PJIs). Among the sample types evaluated, sonication fluid cultures demonstrated the highest diagnostic accuracy, with a pooled sensitivity of 78%, specificity of 91%, and an area under the receiver operating characteristic curve of 0.90, highlighting their effectiveness in identifying biofilm-associated infections (160). In another meta-analysis regarding the use of sonication for biofilm-related infections of cardiovascular implantable electronic devices (CIEDs), Martín Gutiérrez et al. evaluated nine studies (1,684 cultures) comparing sonicated vs. nonsonicated CIED cultures. The authors reported that although the sensitivity of cultures following sonication was increased, there was also an increase in false-positive results with sonication, limiting its specificity (161). The same concerns regarding the lower specificity of sonicated cultures were raised in another meta-analysis by Araujo et al., which evaluated the diagnostic accuracy of cultures following sonication of CIED explants (162). The authors of this study reported that the specificity of sonicated cultures was only 63%. This lower specificity may be due to the detection of minute, non-pathogenic contaminants introduced during surgery, explant handling, or processing. Overall, sonication is a valuable adjunct tool that can improve biofilm pathogen detection and should be considered, especially if prior cultures are negative in patients with high clinical suspicion of biofilm infection. However, the results of sonicated cultures should be interpreted with caution, along with clinical context, since enhanced sensitivity comes with some specificity trade-offs. Optimal protocols using specific threshold settings, along with other techniques such as matrix-assisted laser desorption ionization time-of-flight (MALDI-TOF), may increase the reliability of the obtained results.

In contrast to planktonic bacteria, which can be cultivated in routine media, biofilm bacteria may not be culturable (163–165). In cases where biofilm bacteria are not culturable (e.g., *L. pneumophila*), genetic sequencing techniques can enhance diagnostic outcomes by detecting bacterial DNA/RNA directly from clinical samples (166). Genetic sequencing techniques include polymerase chain reaction (PCR)-based assays, next-generation sequencing (NGS), and transcriptomics (150, 167, 168). 16S rRNA PCR is a widely used PCR-based assay using primers toward the 16S ribosomal RNA gene, a genetic region that is present in all bacteria. This gene also contains species-specific parts; thus, the result of the sequenced genetic material can be compared to databases to identify the bacterial species. Although the sensitivity of 16S rRNA PCR is improved on implant sonicate fluid or tissue samples, many studies evaluating 16S rRNA PCR in non-sonicated fluid samples have reported a similar sensitivity rate compared to conventional cultures, of about 70%–80% (169, 170). Several reasons may explain this, such as the fact that bacteria in biofilms may shed only minimal DNA in the surrounding fluids, especially when standard sample processing does not disrupt the biofilm sufficiently, or when ultra-clean techniques to avoid contamination are selected by applying conservative DNA thresholds. Moreover, the limited number of used primers, especially of those targeting species-specific regions, and the curated databases that are used to match sequences to the species level may decrease the identifying capacity of 16S rRNA PCR. These limitations can be overcome by NGS, a technique that sequences all genes simultaneously and maps them against reference databases. NGS, as opposed to 16S rRNA PCR, can detect multiple organisms simultaneously, even in cases of rare or unexpected pathogens, while it also provides information on resistance genes. In orthopedics, several recent studies have evaluated the diagnostic value of NGS for PJI across different samples, including synovial fluid, prosthetic sonicate fluid, and periprosthetic tissue, reporting a pathogen detection rate of 90%–95%, with sonicate fluid yielding the best results (171, 172). Similar to orthopedics, NGS adds diagnostic value in both localized and systemic CIED infections, outperforming culture in many cases, especially when device material is analyzed (173). Transcriptomics refers to the genetic sequencing of mRNA, providing additional information on the functionality and activity of the involved pathogens by analyzing the differential expression of the bacterial genes. This is especially important in biofilms, since it has been shown that certain changes in bacterial gene expression are associated with the switch from the planktonic phase to the biofilm phase. This technology is currently used in research settings, since it is mostly used for the evaluation of the bacterial growth dynamics in biofilms, rather than for the detection of the presence of pathogens in a clinical sample (157, 174). Conclusively, NGS is currently the most promising genetic sequencing technique, especially helpful in cases of culture-negative and polymicrobial infections, or when previous antibiotics have been used that may have suppressed culture and PCR yield. Moreover, NGS provides resistance profiling, which is crucial for managing biofilm-associated infections. However, routine use of NGS is currently under evaluation in biofilm-associated infections, since there are certain drawbacks with its universal application. NGS is especially prone to false-positive results due to contamination, has a higher cost while it cannot differentiate between live and dead pathogens. This can lead to overdiagnosis or misinterpretation of clinically irrelevant DNA, especially in chronic infections or post-antibiotic samples.

Although genetic sequencing techniques provide valuable information regarding the bacterial genome and the included genes, this does not equate to which genes are translated into proteins, and how the products of these genes are utilized in biofilm networks. This can be done by proteomics, an emerging technology that refers to the analysis of the proteins that are present in a sample. Proteomics in biofilms can not only aid in the identification of the causative pathogens, but also inform about key proteins that are important in biofilm structures, such as proteins essential for temperature adaptation, or proteins contributing to the switch from planktonic to biofilm phase (175, 176). Most proteomic studies use different methodologies of mass spectrometry, with MALDI-TOF mass spectrometry (MS) being an increasingly valuable proteomic study for diagnosing biofilm-associated infections (150, 177). MALDI-TOF MS uses a laser pulse on intact bacteria or bacterial extracts, resulting in evaporation of microbial proteins into ionized particles of a certain charge. The mass-charge ratio is compared to a reference library of known organisms, leading to pathogen identification (178). This technique provides rapid species-level identification. Although this technique has already demonstrated clinical utility when rapid identification of bacteria is needed, such as in the management of IE (179), and shows promise for directly detecting bacteria from positive blood culture bottles or cerebrospinal fluid samples, there are certain limitations with its use in biofilm-related infections (180, 181). Its sensitivity is limited in polymicrobial infections, a common scenario in biofilm infections, where it can identify the most abundant pathogen or cannot detect any pathogen at all (182). When multiple pathogens are present in a clinical sample, the resulting aberrant peptide spectrum is a mixture of several profiles, which is not identifiable as a certain microorganism. Based on recent studies, the sensitivity of MALDI-TOF of synovial or sonicated fluid for diagnosis of PJI is comparable to tissue or fluid cultures (183, 184). Moreover, challenges such as complex sample preparation, need for substantial financial investment, biofilm variability, the necessity for extensive reference databases, and lack of technical expertise continue to limit widespread clinical implementation (150, 178, 185). Conclusively, the main advantage of MALDI-TOF is the speed at which it provides positive results, enabling quicker targeted therapy. However, it should be best used alongside, not instead of, conventional culture methods, since it can miss certain cases of polymicrobial infections or slow growers like *C. acnes*.

Overall, methods like tissue culture, sonicated fluid culture, and genetic sequencing techniques currently dominate clinical diagnostics of biofilm infections due to their practicality, cost-effectiveness, and proven reliability. In 2022, the updated version of the (ESCMID 2014) guidelines reaffirms that the 2014 guidelines, despite new findings, remain applicable for clinical practice. At the same time, it acknowledges that standardized measures for the diagnosis of bacterial biofilms have yet to be established and points out the urgent need for in-depth research aiming at validated diagnostic procedures (100, 186).

## RECENT ADVANCES IN TREATMENT

**Fig 2:**
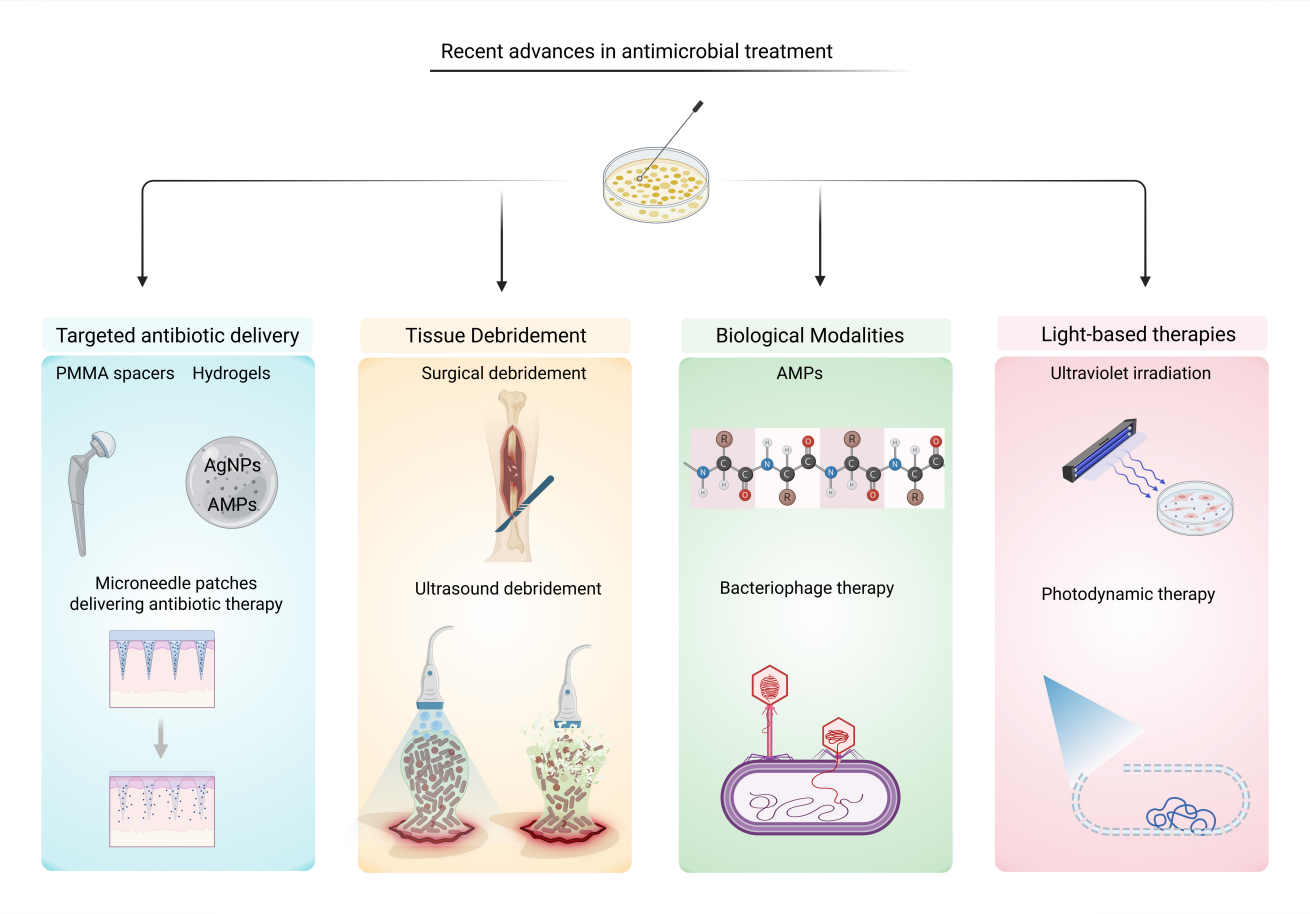
Recent advances in antimicrobial treatment strategies. Approaches include the following: Targeted antibiotic delivery (e.g., polymethylmethacrylate (PMMA) spacers, hydrogels, microneedle patches), tissue debridement (surgical and ultrasound-assisted methods), biological modalities (antimicrobial peptides [AMPs], bacteriophage therapy), and light-based therapies (ultraviolet irradiation and photodynamic therapy). AgNPs: silver nanoparticles. Created in BioRender (2025) https://BioRender.com/bjxc4gv*.*

Biofilm-associated infections represent a persistent and complex challenge in clinical medicine due to their high resistance to antibiotics and immune clearance. Recent research has led to innovative treatment strategies that target biofilm structure, enhance drug delivery, and modulate host-pathogen interactions. This section highlights some of the most promising advancements in biofilm therapy (Fig. 2).

A major challenge in treating biofilm-associated infections with antibiotics is delivering sufficient drug concentrations at the site of infection. Biofilms often require antibiotic levels that are 100–800 times higher than those needed to inhibit planktonic bacteria, necessitating prolonged high-dose and combination therapies to achieve effective eradication (187). In IBC-associated *E. coli* UTI, the dispersal phase involves bacterial filamentation and escape into the bladder lumen and creates a “planktonic window” that may be more susceptible to antibiotics (188, 189). The increased resistance is attributed to multiple factors, including the protective extracellular matrix, reduced metabolic activity of bacteria within the biofilm, the presence of persister cells, and also the ability of bacterial genomes to rapidly adjust in response to environmental changes (8, 190). Importantly, the polymicrobial nature of many biofilms further complicates treatment (191). Interspecies interactions within these complex communities can enhance antimicrobial tolerance, as certain species may produce protective enzymes or signaling molecules that promote resistance mechanisms in neighboring organisms (192, 193).

Given these challenges, delivering antibiotics directly to the infection site has become a key strategy to improve treatment outcomes. Targeted antibiotic delivery to infection sites enhances treatment of biofilm-related conditions by achieving high local drug concentrations while minimizing systemic toxicity. Antibiotic-loaded PMMA spacers are widely used to suppress infection and prevent biofilm regrowth in PJIs (194). These spacers are commonly used in two-stage revision procedures, where an infected implant is removed and temporarily replaced with a drug-loaded spacer (195). However, PMMA is non-biodegradable, requires removal, and may be prone to colonization once antibiotic levels drop. To address these limitations, biodegradable alternatives like calcium sulfate beads and hydrogels are being explored for their ability to release antibiotics more consistently and eliminate the need for a second surgery (196–198). Recent clinical data show infection control rates above 60% in the first year in acute periprosthetic joint infections treated with antibiotic-loaded calcium sulfate beads (199).

Hydrogels embedded with antimicrobial agents, such as silver nanoparticles (AgNPs) and AMPs (200–202), have demonstrated effective disruption of biofilms while maintaining biocompatibility. For instance, AgNPs integrated into hydrogel matrices have shown potent antibacterial activity against various pathogens, including *Staphylococcus aureus* and *Escherichia coli*, without significant cytotoxic effects (203). Expanding on this approach, hydrogels have also been utilized as delivery systems for conventional antibiotics. Biodegradable thermosensitive hydrogels loaded with daptomycin have shown effective *S. aureus* biofilm reduction *in vitro*, indicating potential for treating implant-associated infections (204). Complementing hydrogel-based systems, microneedle patches have emerged as a promising approach for localized antibiotic delivery, particularly for the treatment of accessible skin and soft tissue infections (205). Xu et al. demonstrated that microneedle patches loaded with nanoparticle-encapsulated antibiotics are capable of penetrating biofilm structures, with drug release specifically triggered by bacterial enzymes, resulting in effective biofilm disruption (206). Separately, Chen et al. highlighted the broader potential of microneedles to improve transdermal drug delivery for a range of cutaneous conditions, including bacterial infections (207). These minimally invasive systems offer localized, sustained antimicrobial delivery with reduced risk of systemic side effects.

Surgical debridement remains a cornerstone in the management of chronic wounds complicated by biofilm formation, primarily through the removal of necrotic tissue and reduction of microbial burden to support wound healing. However, its invasiveness, potential for pain, and the risk of promoting deeper tissue colonization by residual biofilm microcolonies present significant limitations (208, 209). To address these limitations, ultrasound debridement has emerged as a less invasive alternative. By employing low-frequency ultrasonic waves, this technique effectively disrupts biofilms, facilitates tissue regeneration, and is associated with reduced patient discomfort and inflammation (210–212). Studies indicate that ultrasound debridement can enhance healing rates in chronic wounds, such as diabetic foot ulcers and venous leg ulcers, by effectively diminishing biofilm presence and bacterial burden (213, 214). Clinical trials report up to 36% higher complete healing rates with ultrasound-assisted therapy compared to standard debridement (215).

As conventional treatments often fall short in eradicating biofilms, several innovative strategies are being investigated. AMPs offer a broad-spectrum approach by disrupting bacterial membranes and interfering with genes critical to biofilm formation; however, resistance to AMPs remains a concern (216). Furthermore, a significant challenge to their clinical application in the treatment of systemic infections is their susceptibility to rapid enzymatic degradation, resulting in markedly reduced bioavailability (217–219). Bacteriophage therapy represents another emerging strategy that employs viruses, or the lytic enzymes they produce, to specifically target and eliminate bacteria, including those residing within biofilms (220, 221). By degrading the biofilm’s protective extracellular matrix, these enzymes enhance bacterial clearance and improve treatment efficacy (222). They can be used alone or in combination with antibiotics to improve efficacy (223, 224). Phage-antibiotic combinations have achieved favorable outcomes; clinical improvement in ~77% and eradication in ~61% of cases in a cohort of 100 difficult-to-treat infections (225). Shifting from biological to physical modalities, light-based therapies—such as ultraviolet irradiation and photodynamic therapy—exert their antimicrobial effects by damaging microbial DNA or generating reactive oxygen species (226–228). While effective on surface-level infections, their clinical utility is limited by poor light penetration and potential host tissue damage. In 90 patients with chronic infected wounds, photodynamic therapy achieved clinical improvement in 84% of cases (229).

The advent of novel therapeutic strategies targeting biofilm-associated infections has outpaced the diagnostic tools that are traditionally used to guide antibiotic therapy. The traditional antimicrobial susceptibility testing (AST) methods, such as disk diffusion or automatic broth microdilution methods, evaluate free-living bacterial populations without taking into account the complexity and resilience of biofilms. The emerging biofilm-directed therapies—including AMPs, phage-based agents, AgNPs, and localized delivery systems such as hydrogels and microneedle patches—employ mechanisms that cannot be measured from standard AST protocols. The goal of these approaches is to overcome systemic pharmacokinetic limitations by disrupting biofilms, enabling enzyme-mediated drug release, or delivering high antimicrobial concentrations locally. As a result, the minimum inhibitory concentrations (MICs) of antibiotics that are obtained from planktonic cells and under standard testing conditions cannot be used to predict treatment outcomes in biofilm-associated infections.

In 1999, Ceri et al. were the first to propose the concepts of minimum biofilm inhibitory concentration (MBIC) and minimum biofilm eradication concentration (MBEC), which are defined as the minimal concentration of antibiotics required to inhibit or eradicate a biofilm (230). In their study, the researchers introduced the Calgary Biofilm Device (CBD)—a 96-well microtiter plate equipped with a lid containing removable pegs. To encourage bacterial attachment to the pegs, the wells are gently shaken after being inoculated with broth and bacteria. During the incubation period, biofilms develop on the surface of the pegs, while planktonic cells remain suspended in the broth (231). Since then, different modifications based on the CBD system have emerged, as well as other approaches such as flow cell systems, which enable real-time imaging of biofilm development under dynamic conditions, and various biofilm quantification assays, which assess the metabolic activity of detached cells as an indicator of their viability in response to antimicrobial treatment (231–234). Nevertheless, these methods are not yet standardized for routine clinical use, biofilm-specific breakpoints for MBIC and MBEC have not yet been established, and the lack of regulatory validation hinders a widespread implementation of these assays.

Over the past decade, significant progress has been made in understanding the pathophysiology of biofilms, leading to the development of innovative therapeutic strategies. Among these, localized antibiotic delivery and ultrasound-assisted debridement have shown the strongest evidence of clinical efficacy. Phage therapy, AMPs, and nanoparticle-based systems remain largely experimental but provide promising evidence for future clinical translation. These approaches aim to overcome the limitations of traditional therapies. Bridging the gap between therapeutic innovation and diagnostics is now more necessary than ever, since it could help bring next-generation treatments sooner into clinical use.

## SUMMARY AND FUTURE PERSPECTIVES

Biofilms are now recognized as a major cause of chronic and device-related infections, changing how we think about diagnosis and treatment. As we learn more about how biofilms form and survive, the way we approach microbiology is evolving. New diagnostic tools—like sonication, advanced imaging techniques, NGS, and mass spectrometry—can yield higher sensitivity and broader pathogen detection than traditional cultures. Treatments are also evolving beyond high-dose antibiotics and surgery to include newer methods like hydrogels, microneedle patches, peptides, and phage therapy. Despite this progress, major gaps remain. Diagnostic tools with high precision, as well as promising therapeutic innovations, are often unavailable in routine clinical settings. Current treatment standards still focus on planktonic bacteria and fail to address the resilient and polymicrobial nature of biofilms. Future efforts must prioritize the translation of research into standardized clinical protocols. This includes a broader implementation of molecular diagnostics and a systematic clinical validation of novel treatments. Finally, clinical guidelines should be updated to incorporate the biofilm model of infection.
